# Preliminary survey on *Anopheles* species distribution in Botswana shows the presence of *Anopheles gambiae* and *Anopheles funestus* complexes

**DOI:** 10.1186/s12936-017-1756-5

**Published:** 2017-03-07

**Authors:** Leabaneng Tawe, Pleasure Ramatlho, Kelebogile Waniwa, Charles W. Muthoga, Ntebaleng Makate, Davis S. Ntebela, Isaac K. Quaye, Marco Pombi, Giacomo Maria Paganotti

**Affiliations:** 1 Department of Medical Laboratory Sciences, Faculty of Health Sciences, University of Botswana, Gaborone, Botswana; 2 Botswana-University of Pennsylvania Partnership, PO Box AC 157 ACH, Gaborone, Botswana; 3 National Malaria Control Programme, Botswana Ministry of Health, Gaborone, Botswana; 4 Department of Biological Sciences, Faculty of Science, University of Botswana, Gaborone, Botswana; 5 Department of Biochemistry, University of Namibia School of Medicine, Windhoek, Namibia; 6 Department of Public Health and Infectious Diseases, “Sapienza” University of Rome, Rome, Italy; 7 Perelman School of Medicine, University of Pennsylvania, Philadelphia, PA USA; 8 Department of Biomedical Sciences, Faculty of Medicine, University of Botswana, Gaborone, Botswana

**Keywords:** *Anopheles gambiae*, *Anopheles funestus*, Botswana, Malaria, *Plasmodium falciparum*

## Abstract

**Background:**

Botswana is one of the four front line malaria elimination countries in Southern Africa, with malaria control activities that include routine vector control. Past and recent studies have shown that *Anopheles arabiensis* is the only known vector of *Plasmodium* parasites in the country. This report presents a preliminary evaluation on *Anopheles* species composition in seven districts of Botswana with some inferences on their vectorial role.

**Results:**

Overall, 404 *Anopheles* mosquito females were collected, of which 196 were larvae collected from several breeding sites, and 208 were adults obtained from indoor pyrethrum spray catches (PSC). *Anopheles arabiensis* (58.9%) accounted for the highest relative frequency in 5 out of 7 districts sampled. The other species collected, among those identified, were barely represented: *Anopheles longipalpis* type C (16.3%), *Anopheles parensis* (8.9%), *Anopheles quadriannulatus* (5.4%), and *Anopheles leesoni* (0.2%). PCR test for human β-globin on mosquitoes collected by PSC showed that *An. arabiensis* and *An. parensis* had bitten human hosts. Moreover, *An. arabiensis* showed a non-negligible *Plasmodium falciparum* infection rate in two sites (3.0% and 2.5% in Chobe and Kweneng West districts, respectively).

**Conclusions:**

This work provides first time evidence of *Anopheles* diversity in several areas of Botswana. *Anopheles arabiensis* is confirmed to be widespread in all the sampled districts and to be vector of *P. falciparum*. Moreover, the presence of *Anopheles funestus* group in Botswana has been assessed. Further research, entomological surveillance activities and possibly vector control programmes need to be better developed and implemented as well as targeting outdoors resting vectors.

## Background

Botswana is one of the four Southern African countries on the nearing malaria elimination (together with South Africa, Namibia and Swaziland). Therefore, knowledge of the transmission dynamics is critical in moving forward. Malaria mainly occurs in five Northern and Eastern districts (Okavango, Ngami, Chobe, Boteti and Tutume) with other districts being affected occasionally due to local outbreaks/epidemics, because the country’s ecosystem is receptive to malaria [1]. These regions experience active malaria circulation, especially during the peak of malaria vector breeding season that spans the summer months (November–April). Each of these districts have developed malaria control programmes, including routine vector control, which is primarily based on the application of indoor residual insecticide spraying (IRS) and use of *long*-*lasting* insecticide-treated nets (*LLINs*) [2]. In Botswana, the only known malaria vector is *Anopheles arabiensis*, a mosquito belonging to the *Anopheles gambiae* complex known to be a major vector of *Plasmodium falciparum* and *Plasmodium vivax* parasites. The presence of *An. arabiensis* as the main malaria vector in Botswana has been recently assessed on the Okavango region in North-West Botswana [3–5]. No further information is available from other areas of the country. Moreover, nothing is known about the presence of other species, such as those of the *Anopheles funestus* complex, which includes major malaria vector species of Southern Africa [6–8].

In general, several of these species are often found to occur in sympatry and their importance in malaria transmission varies depending on behaviour, seasonal prevalence and vectorial capacity. These peculiarities contribute to the varied malaria epidemiological patterns observed in a particular geographical region. Therefore, accurate identification of malaria vector system in a defined area is important in the understanding of transmission dynamics scenario. There is a clear need for Botswana to carry out vector surveillance studies in order to obtain baseline knowledge on seasonal prevalence and vectorial capacity in areas where malaria is of unstable endemicity. This can help in understanding the malaria vectorial system and, therefore, identify the risk areas and the local foci of potential transmission where malaria could be re-introduced. It is important to point out that Botswana has high vulnerability to malaria due to the fact that rainfall anomalies are widely considered to be a major driver of inter-annual variability of malaria incidence in particular in semi-arid areas of Southern Africa, following the El Nino Southern Oscillation (ENSO) pattern [2, 9, 10].

The aim of this work is to present the spectrum of *Anopheles* species composition of specimen collected in 2015 from several districts of Botswana, where malaria is of unstable endemicity, and from one Southern district at the time of a malaria outbreak in 2012. For some areas presented here, this is the first report of malaria vectors presence in recent years. Moreover, the information on the detection of human blood and the *Plasmodium* positivity rates in the specimens collected is provided.

## Methods

**Fig. 1:**
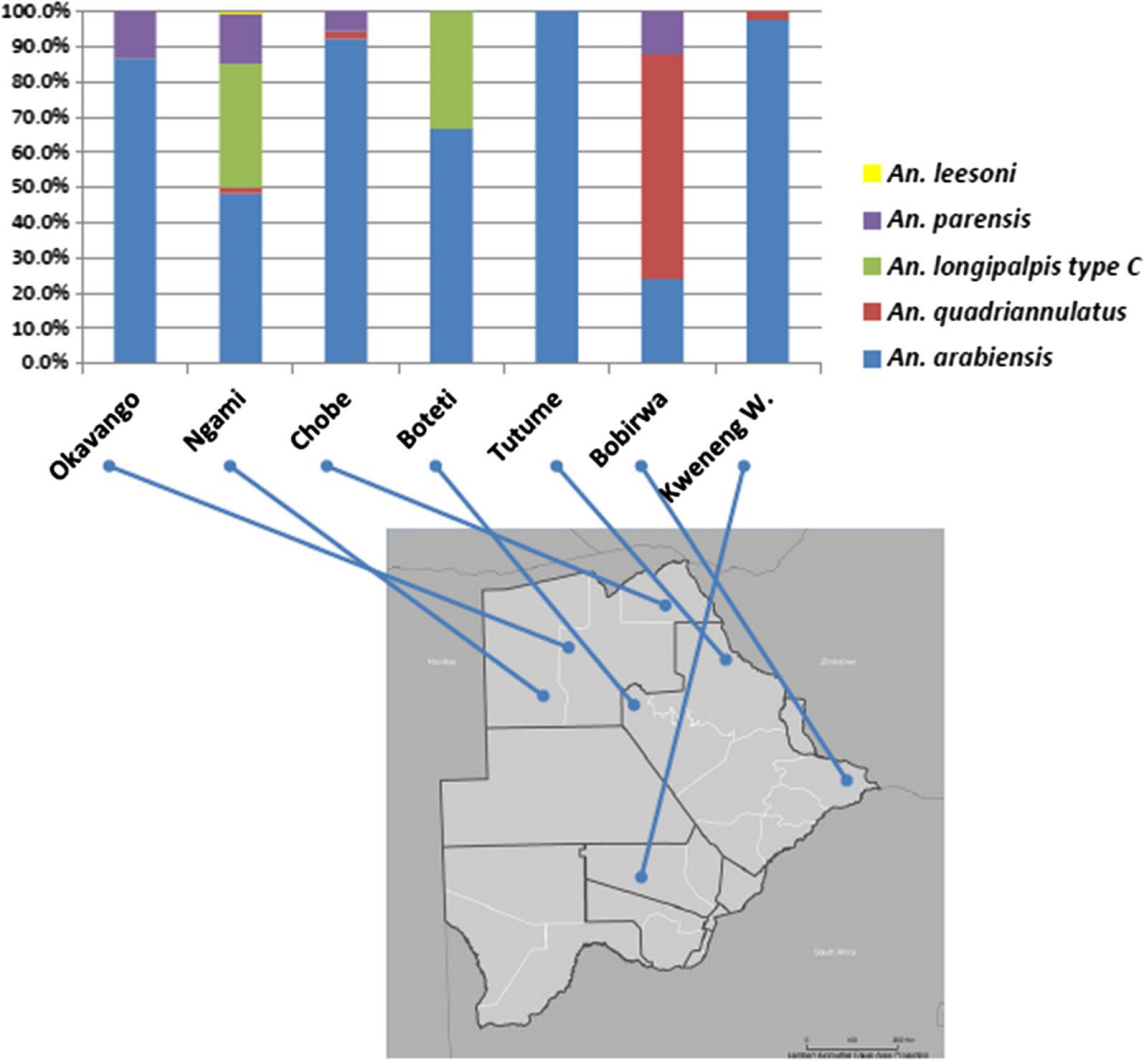
*Anopheles* species composition and geographical distribution in 7 districts of Botswana

*Anopheles* specimens were collected in 7 districts of Botswana (Kweneng West in March 2012; Okavango, Ngami, Chobe, Boteti, Tutume, Bobirwa in February–March 2015) (see Fig. 1). In 2011, seven sentinel sites were established to develop a surveillance system for malaria vector population and monitor vector susceptibility to insecticides. The sites represent three epidemiological zones: A-endemic malaria transmission (Okavango, Ngami, Chobe), B-moderate malaria transmission (Boteti, Tutume, Bobirwa), C-malaria free zones but prone to outbreaks (Kweneng West) [11]. Entomological surveillance was done in those 7 sites to provide insight information on vector density, monitor vector susceptibility to insecticides and assess quality of vector control interventions (IRS/LLINs). The locations were chosen after assessing the availability of potential breeding sites for mosquito sampling for each location (with radius of 2–10 km). Mosquitoes were collected both as larvae from several breeding sites and as resting adults by indoor Pyrethrum Spray Catches (PSC). The PSC were performed for 2–3 days in each site and 40 houses per site were sampled. Collected larvae were brought to the insectary and allowed to emerge as adult before morphological and genetic analysis was performed. Adult female specimens were first identified morphologically [12], before molecular identification in case of specimens belonging to sibling species complexes.

The protocol of Scott et al. [13] was applied for the species identification of *An. gambiae* sensu lato (s.l.) specimens and the protocol of Koekemoer et al. [6] for species identification of those belonging to *An. funestus* s.l. complex. Finally, to further identify the presence of *Anopheles longipalpis* type C in the *An. funestus* s.l. samples, the protocol of Choi et al. [14] was adopted.

As no information was taken on the gonotrophic stage of the specimens collected as adults, all specimens were tested for the presence of human blood through the detection of human β-globin DNA specific sequence, according to the protocol of Quinones et al. [15]. Moreover, the same specimens were also tested for the presence of *P. falciparum* using the molecular detection for *pfmdr1* parasite gene through a nested-PCR approach [16]. For both tests we used appropriate positive controls (human DNA, and HB3 and DD2 *P. falciparum* parasite strains, respectively).

## Results

Four hundred and four (404) female *Anopheles *mosquitoes were collected in seven districts of the country, as shown in Table 1. Of these, 196 were larvae collected from several breeding sites, and 208 were adults obtained by indoor PSC. Forty one (41) out of 404 mosquitoes were not identified both morphologically and by molecular analysis because of the bad preservation status of the specimens (identification success rate 89.9%). In 3 out of 7 districts (Boteti, Tutume, Bobirwa) the proportion of unidentified specimens varied from 31 to 50% (Table 1). There was a large variability of species collected in the different sites (Fig. 1), although *An. arabiensis* (58.9%), was identified with higher relative frequency in 5 out of 7 districts. The other species collected, among those identified, were far less represented: *Anopheles quadriannulatus* (5.4%) which is a non-vector species belonging the *An. gambiae* complex and *An. longipalpis* type C (16.3%), *Anopheles parensis* (8.9%) and *Anopheles leesoni* (0.2%) all belonging to the *An. funestus* complex. The adult mosquitoes collected by PSC and tested for human blood showed that *An. arabiensis* and *An. parensis* had bitten human hosts (Table 2). In addition, *An. arabiensis* showed a noticeable *P. falciparum* infection rate in two sites (3.0% in Chobe and 2.5% in Kweneng West, Table 3), with one single mosquito found to be positive to parasite DNA in both sites.

**Table 1:** Scheme of *Anopheles* mosquitoes collected in the different districts sampled

District of collection	Village	Coordinates	Method of collection	*An. arabiensis*	*An. longipalpis C*	*An. parensis*	*An. quadriannulatus*	*An. leesoni*	Unidentified	Total
Okavango	Mohembo East	S 18° 15′ E 21° 47′	PSC	8		2				10
	Mohembo West	S 18° 17′ E 21° 47	PSC	3						3
	Mogotlho	S 19° 13′ E 23° 57′	PSC	2						2
Ngami	Ditshiping	S 19° 46′ E 23° 25′	PSC	21	2	3				26
	Shorobe	S 19° 45′ E 23° 40′	LC	36	34	12	3	1	8	94
			PSC	32	29	11			2	74
Chobe	Kavimba	S 18° 04′ E 24° 34′	LC	50		5	1		15	71
	Mabele	S 17° 59′ E 24° 38′	PSC	33			1			34
Boteti	Motopi	S 20° 13′ E 24° 08′	PSC	2	1				3	6
Tutume	Nata	S 20° 12′ E 26° 10′	PSC	4					2	6
Bobirwa	Mathathane	S 22° 16′ E 28° 44′	LC	4		3	13		11	31
			PSC	2			3			5
Kweneng West	Khudumelapye	S 23° 52′ E 24° 45′	PSC	41			1			42
Total				238	66	36	22	1	41	404

*PSC* pyrethrum spray catch, *LC* larval collection

**Table 2:** Human blood positivity in PSC *Anopheles* species

District of collection	Village	Human biting			
		*An. arabiensis* (% of human DNA positivity)	*An. quadriannulatus* (% of human DNA positivity)	*An. longipalpis* type C (% of human DNA positivity)	*An. parensis* (% of human DNA positivity)
Okavango	Mohembo East	37.5	–	–	0.0
	Mohembo West	33.3	–	–	–
	Mogotho	100.0	–	–	–
Ngami	Ditshiping	9.5	–	0.0	0.0
	Shorobe	0.0	–	0.0	27.3
Chobe	Mabele	6.1	0.0	–	–
Boteti	Motopi	50.0	–	0	–
Tutume	Nata	25.0	–	–	–
Bobirwa	Mathathane	0.0	0.0	–	–
Kweneng West	Khudumelapye	20.0	0.0	–	–
Total number of mosquitoes identified		148	5	32	16

**Table 3:** *Plasmodium falciparum* positivity rate in PSC *Anopheles* species

District of collection	Village	*Pf* infection rate			
		*An. arabiensis* (% of *Plasmodium falciparum* DNA positivity)	*An. quadriannulatus* (% of *P. falciparum* DNA positivity)	*An. longipalpis* type C (% of *P. falciparum* DNA positivity)	*An. parensis* (% of *P. falciparum* DNA positivity)
Okavango	Mohembo East	0.0	–	–	0.0
	Mohembo West	0.0	–	–	–
	Mogotho	0.0	–	–	–
Ngami	Ditshiping	0.0	–	0.0	0.0
	Shorobe	0.0	–	0.0	0.0
Chobe	Mabele	3.0 (1 out of 33)	0.0	–	–
Boteti	Motopi	0.0	–	0.0	–
Tutume	Nata	0.0	–	–	–
Bobirwa	Mathathane	0.0	0.0	–	–
Kweneng West	Khudumelapye	2.5 (1 out of 40)	0.0	–	–
Total		148	5	32	16

## Discussion

In this report, we provide information about *Anopheles* diversity in several areas of Botswana, covering the Northern to the Southern parts of the country. The results, although with relatively small sample size, confirm the widespread presence of *An. arabiensis* in all the sampled districts. Diversity higher than previously reported [17] was observed, and in particular a noticeable presence of some species of the *An. funestus* group, which are unknown or potential vectors of *Plasmodium* parasites [18]. Furthermore, the presence of human DNA in indoor resting *An. arabiensis* and *An. parensis* in several districts was detected, and it was confirmed the possible vector role of *An. arabiensis* for *P. falciparum* in Chobe and Kweneng West districts. In fact, the *P. falciparum* infected *An. arabiensis* from Chobe district was not positive for human β-globin DNA, supporting the hypothesis that the sporogonic cycle of the malaria parasite was not at an early stage (blood meal digestion), but progressed towards oocyst formation or salivary glands invasion [19, 20]. Instead, for the *An. arabiensis* positive for *P. falciparum* and human β-globin DNA from Khudumelapye (Kweneng West) it is possible to infer either a recent *P. falciparum*-positive blood meal (with or without gametocytes) or a possible second blood meal of an already infected mosquito.

In general, the data does not provide any information about anthropophily of the species collected, but confirm that humans are among the hosts of indoor resting *An. arabiensis* and *An. parensis* in Botswana. While the role of *An. arabiensis* as malaria vector is generally known, this paper presents some evidence of anthropophagic behaviour of *An. parensis* in Botswana. Moreover, the latter data is supported by the record of *P. falciparum* infected specimens of *An. parensis* species in neighbouring South Africa [21].

*Anopheles arabiensis* populations exhibit both exophilic and endophilic behaviours and various degrees of anthropophily depending on the prevailing ecological conditions [22]. This has implications for the planning of vector control measures. Although in Botswana it has been broadly proclaimed that malaria transmission is mostly due to *An. arabiensis*, it is not known how the transmission process plays out (indoor or outdoor transmission). This is important in the selection of the most effective tools for combating the vector which indirectly affects the effectiveness of control measures for blocking transmission. Moreover, after more than 70 years of indoor residual spraying in the Botswana [23], it could be that indoor biting behaviour of the *An. arabiensis* population may have shifted from indoor to outdoor biting preference with possible impact on malaria transmission, as already observed elsewhere [24, 25]. Recently, a genetic component linked to the biting behaviour has been postulated [26], congruently with the hypothesis that a possible shift toward a higher degree of zoophily in *An. arabiensis* could be due to selection by indoor vector control activities. Conversely, insecticide resistance could have played an opposite role in selecting for insecticide resistant populations, thus negatively impacting on indoor residual spraying activity and ultimately in malaria control.

Unfortunately, Botswana lacks almost all data on local vector species and their susceptibility to insecticides, as well as on vector and human behaviours that may allow vectors to avoid contact with interventions and then maintain residual transmission. There is also a critical need for punctual monitoring of the coverage, usage, quality and durability of vector-control interventions such as IRS and *LLINs*. Evaluation of the impact of interventions on malaria outcomes should also be undertaken. Moreover, Botswana urges to invest better in public health entomology capacity, to support the control and elimination of malaria [27].

Despite the malaria elimination campaigns, Botswana still experiences a significant number of reported cases with deaths [28]. In the malaria elimination setting it is crucial to know where to target the interventions. In Botswana interventions are primarily focused on vector control but clearly additional measures, especially those targeting outdoor resting vectors, need to be evaluated. Entomological surveillance activities, including routine insecticide resistance monitoring, need to be scaled up in order to prevent new outbreaks driven by resistant and new vector populations.

## Conclusion

This study provides a first report on the spread and abundance of *An. gambiae* and *An. funestus* complex in Botswana. Additionally, there is evidence of *An. arabiensis* as malaria vector in Botswana and a possible potential role of *An. parensis*. Future strategies on vector control must take into consideration more tools that target both indoor and outdoor transmission of *Plasmodium* species.
